# Safeguarding Online Research in Eating Disorders Against Fraud: Increasing Risks and Practical Recommendations

**DOI:** 10.1002/eat.70083

**Published:** 2026-03-31

**Authors:** Jamie‐Lee Pennesi, Mia L. Pellizzer, Tracey D. Wade

**Affiliations:** ^1^ Flinders University Institute for Mental Health and Wellbeing and Blackbird Initiative, Flinders University Adelaide South Australia Australia

**Keywords:** digital research, eating disorders, fraud, fraudulent participants, Internet research, online research

## Abstract

**Objective:**

Recent growth of online research has been accompanied by an increase in reports of fraudulent participants, which can significantly comprise research validity. Drawing from our experience using *Qualtrics* with open recruitment, existing literature, and emerging studies in eating disorders (ED), we outline the risk and provide simple, practical recommendations for preventing, detecting, and managing fraudulent participants in online ED research.

**Method:**

Over the conduct of a three‐round Delphi consensus study with 138 English‐speaking individuals aged 18 and older, we were inundated with fraudulent sign‐ups between July and August 2024, despite implementing multiple fraud prevention strategies. In response, we introduced additional fraud mitigation strategies and established a three‐step procedure for identifying and managing fraudulent participants.

**Results:**

The additional fraud mitigation measures, including a second reCAPTCHA, a duplicate question for consistency checks, and modified attention check questions, potentially aided in preventing further fraudulent sign‐ups. Our procedure involving manual comprehensive review of all incoming survey data and checks against a fraudulent participants' profile enabled us to identify and withdraw suspected or likely fraudulent participants.

**Discussion:**

With increasing fraudulent participation rates and rapidly advancing technological advancements such as artificial intelligence, all online studies are at risk and researchers need to be proactive in their use of antifraud practices to safeguard online research. Our practical recommendations can assist future researchers in managing fraudulent participants.

## Summary

- Fraudulent participation in online research is rising rapidly and presents a significant threat to research integrity.
- All online studies are at risk and researchers must adopt proactive antifraud practices to safeguard data quality.
- We provide straightforward, practical recommendations for preventing, detecting, and managing fraudulent participation in online research.

## 1 Introduction

### 1.1 Online Research and Its Benefits

The popularity of online research, including surveys and the use of crowdsourcing websites such as Amazon's Mechanical Turk (MTurk), has exploded in recent decades (Evans and Mathur 2018; Hulland and Miller 2018; Lawlor et al. 2021; Wright 2005; see Gosling and Mason 2015 for a review). This coincides with increased use of digital devices and networks such as smartphones, widespread adoption of online platforms such as social media (e.g., Instagram, TikTok, X/Twitter), and major advancements in digital technologies such as the development of wearable sensors and artificial intelligence (AI), for example, Chat GPT (Gosling and Mason 2015; Sullivan et al. 2013; Yao and Ling 2020). These online research methods enable access to wider, more diverse populations (e.g., low‐prevalence and hard‐to‐reach groups); recruitment of larger, more representative samples; improved accessibility for participants; flexible research designs (e.g., online features such as branching logic); studies that are time‐ and cost‐efficient, easing burdens on both researchers and participants; and anonymous, unsupervised self‐reporting of responses, encouraging more honest responses by minimizing stigma (Evans and Mathur 2018; Gosling and Mason 2015; King et al. 2014; Wright 2005).

### 1.2 Fraudulent Participants: The Cost of Online Research

The rapid growth of online research has been accompanied by increased reports of fraudulent participants (e.g., Burnette et al. 2022; Goodrich et al. 2023; Lawlor et al. 2021; Roehl and Harland 2022; Storozuk et al. 2020), including individuals or bots (short for robots) deliberately attempting to defraud research studies, commonly to receive financial incentives or influence study results. Bots (also called fraudsters, automatic survey‐takers, automated form‐fillers, or botnets; Buchanan and Scofield 2018; Dupuis et al. 2019; Teitcher et al. 2015) are malicious software applications programmed to perform automated tasks such as filling out online surveys (Griffin et al. 2022; Storozuk et al. 2020; Teitcher et al. 2015). Emerging evidence suggests that advanced agentic AI models, such as the ChatGPT agent (https://openai.com/index/introducing‐chatgpt‐agent/), can complete online surveys in a human‐like manner that may be difficult to distinguish from genuine participants, raising concerns about their potential misuse as automated respondents (Walker et al. 2026). In a 2025 review of fraudulent responses in health research studies employing online recruitment strategies, most published within the last five years, 78% (18/23 studies) reported encountering fraudulent responses (Comachio et al. 2025). Furthermore, another review of 36 online studies shows a significant decline in reports of usable data from 75% to 10% in recent years due to fraudulent participation (Pinzón et al. 2024). Fraudulent participants may retake a screening survey until they learn which responses yield eligibility, sign up to a study multiple times, provide false responses, or deliberately make false claims or exaggerate their experiences to gain access to a study, for example, reporting a certain health condition, geographic location, or gender identity (Roehl and Harland 2022; Saberi 2020; Schneider et al. 2024; Teitcher et al. 2015). In this Forum paper, we focus on fraudulent participation, where research enagement is intentionally deceptive. However, we note that many of the methodological challenges discussed, and several of the potential solutions (e.g., attention checks), also apply to careless or inattentive participants who fail to engage with research questions thoughtfully. Such responding may arise from lack of attention, low effort, or misunderstanding (Bowling et al. 2016; Chandler et al. 2020; Yarrish et al. 2019), but can nonetheless pose similar threats to research integrity (see Ward and Meade 2023, for overview).

Fraudulent participation in online research poses a serious threat to the integrity and validity of research findings by introducing systematic bias, distorting data accuracy (e.g., increasing the risk of Type I and II errors), and potentially invalidating results (Chandler et al. 2020; Dupuis et al. 2019; Gonzalez et al. 2023; Storozuk et al. 2020; Teitcher et al. 2015), a problem detected for quantitative and qualitative studies (Drysdale et al. 2023; Jones et al. 2021; Ménard et al. 2025; Roehl and Harland 2022; Schneider et al. 2024). Fraudulent participants can disrupt targeted recruitment efforts, particularly when specific populations are being studied (e.g., eating disorders [EDs]), and drain valuable resources (e.g., incentives, researcher time). Furthermore, they can also undermine randomization in clinical trials, leading to unbalanced groups that threaten the validity of the findings (Davies et al. 2023). These issues can compromise the effectiveness of subsequent interventions, resulting in the development of interventions lacking validity and may even increase the risk of harm for the intended population.

### 1.3 Strategies for Managing Fraudulent Participation

This phenomenon indicates the urgent need for researchers to be aware of the growing risks and the most effective strategies to prevent and manage them. A rapidly growing body of research is focused on identifying potential strategies for managing fraudulent participation (e.g., Bonnamy et al. 2025; Comachio et al. 2025; Davies et al. 2023; Ménard et al. 2025; Storozuk et al. 2020; Teitcher et al. 2015; Yarrish et al. 2019), advancing statistical methods to detect fraudulent responses in survey data (e.g., Buchanan and Scofield 2018; Dupuis et al. 2019; Irish and Saba 2023; Pinzón et al. 2024), proposing frameworks to guide planning and decision making in study design (e.g., Lawlor et al. 2021; Wessling et al. 2017), and establishing techniques to reliably detect and screen for AI respondents (e.g., Walker et al. 2026), especially as advancing technologies increasingly find ways to outsmart conventional safeguards.

### 1.4 Managing Fraudulent Participation in Eating Disorder Research

The issue of fraudulent participation in online research has been addressed in only a few ED studies; all published within the last four years. The first was Burnette et al. (2022), who raised concerns about the validity of crowd‐sourced data from their experience of MTurk, where 91% of responses were deemed invalid, leading to the project's abandonment. They offered several recommendations to help ED researchers identify and mitigate invalid data. This publication sparked considerable debate among ED researchers regarding the suitability of crowd‐sourcing platforms like MTurk for recruitment and data integrity, the value of conducting online research despite its inherent challenges, and strategies recommended for mitigating fraudulent responding (e.g., De Young and Kambanis 2022; Simone 2022; Vogel et al. 2022).

Davies et al. (2023) was the first to report fraudulent participation within an online clinical trial for early intervention in EDs. Despite having several security measures and a complex design, the study experienced two waves of fraudulent sign‐ups. They outlined the strategies implemented to address these fraudulent attacks, detailing their decision‐making processes and their effectiveness, and provided practical recommendations to future researchers.

Recently, Schneider et al. (2024) reviewed common challenges associated with fraudulent participation in quantitative and qualitative research, drawing on case studies from body image and appearance research, fields that closely align with ED research. They proposed strategies to mitigate these challenges in future research and advocate for a strategic, collaborative, global approach to address the problem. Although fraudulent participation in online research is not unique to ED research, ED studies are published less frequently than those in other areas of mental health (e.g., Frost et al. 2003) and comparatively receive less funding (e.g., Bryant et al. 2023; Murray et al. 2017), despite EDs being among the most severe psychiatric disorders. Consequently, ensuring the validity of data collected online is cruicial for the ED field, where compromised data risks further undermining an already underfunded area. This underscores the relevance and importance of this topic for IJED readers and other peer‐reviewed journals focused on body image and EDs.

### 1.5 Aim of This Forum Discussion

As fraudulent participation continues to rise and traditional prevention methods become less effective amid rapid advances in technologies such as AI, the challenges confronting online research are becoming increasingly complex. Following the helpful structure offered by Davies et al. (2023), the aim of this Forum paper is to provide an overview of the emerging literature across different research areas and provide simple and practical recommendations for preventing, detecting, and managing fraudulent participants, to assist future ED researchers—and researchers broadly—address this issue more effectively. We start by presenting an overview of our experience with fraudulent participants using the platform *Qualtrics* with open recruitment, and describe the evidence‐informed strategies implemented to mitigate this issue. We then provide recommendations for managing these issues in online research, drawing from our experience with fraudulent participants, published research of online detected fraud, and emerging studies in EDs.

## 2 The Delphi Study

### 2.1 Study Design

We conducted a modified three‐round Delphi consensus study with 138 individuals aged 18 and older, who spoke English, and self‐identified as belonging to one of four stakeholder panels (i.e., people with lived experience, significant others, ED clinicians, ED researchers). Participants were recruited online, primarily via the mailing lists, websites, and/or social media pages of reputable ED organizations and research groups, lived experience groups, an ED practitioner's registry, and specialist ED services across Australia. Recruitment was initially limited to Australian residents. Invitations to participate were sent via email, with an overview of the study and a generic hyperlink and QR code linking to the online participant information sheet and consent. The study was described as a project to develop consensus on the essential transdiagnostic targets (a key target of an intervention) to address in future interventions for EDs. Interested participants completed an online screening survey to assess eligibility, and eligible participants were taken directly to the round one survey, which collected demographic information and asked participants to rate the importance of transdiagnostic targets (presented as statements) for inclusion in ED interventions. Participants who completed round one were subsequently invited via email, using unique (personalized) survey links, to rounds two and three, which included a summary of results from the previous round and asked participants to re‐rate statements that had not reached consensus. All surveys were completed online on the survey platform *Qualtrics* between July and September 2024. Completion of each round was required for participation in the next, and completion of all three rounds was required to receive a digital gift card valued at $AUD100. For full details see Pennesi et al. (2025).

### 2.2 Evidence‐Informed Fraud Prevention and Detection Strategies Initially Implemented

Several fraud prevention and detection measures were integrated into the study design from the outset, informed by recent recommendations and firsthand experiences from online studies where fraudulent responses have been detected (e.g., Davies et al. 2023; Lawlor et al. 2021; Roehl and Harland 2022; Storozuk et al. 2020; Teitcher et al. 2015). Most strategies were utilized at multiple timepoints and included: (a) a screening survey; (b) Google revised Completely Automated Public Turing test to tell Computers and Humans Apart (reCAPTCHA) v2 verification; (c) *Qualtrics* in‐built survey features (fraud detection elements e.g., *Prevent multiple submissions, Bot detection*) and analysis of fraud scores; (d) IP address review (i.e., manually checking if country location matched self‐reported postcode); (e) unique (personalized) survey links for subsequent rounds; (f) attention (validity) check questions; (g) open‐ended or free‐text questions; (h) cross‐referenced or duplicate questions; (i) multiple data points; (j) affirming non‐fraudulent participation; (k) targeted recruitment; (l) careful consideration of reimbursement (type, conditions, timing); (m) transparency around fraudulent participation and eligibility for reimbursement; (n) satisficing versus fraud‐generated responses; (o) a fraudulent participants' profile; and (p) regular data quality checks. A detailed summary of these safety measures is provided in Table 1. Given the range and robustness of evidence‐informed strategies in place, we assumed our study was well‐protected against fraudulent participation.

**TABLE 1:** Summary of fraud detection, prevention, and mitigation measures utilized in the current study.

	Strategy^a^	Specific features and applications (with examples)	Timepoint^b^	Category^c^
a.	Screening survey	A screening survey was used to determine eligibility prior to the first‐round survey, which included a mix of yes/no and open‐ended questions, with and without data validation. For example, “Please provide your age in years (e.g., 36)” [number validation], “Do you identify with one of the following groups? … Person with lived experience …” [yes/no], “Please provide your residential postcode (where you currently live)” [text‐entry, no validation].	T0	Prevention, detection
b.	Google reCAPTCHA verification (v2)	The CAPTCHA verification question, an advanced question type in *Qualtrics*, was added to the survey flow. At the time of the study, the v2 CAPTCHA was the default and only option. When participants selected the I am not a robot checkbox, they were asked to complete a challenge (e.g., identify images of traffic lights or crosswalks) before they could proceed with the survey. As recommended by *Qualtrics*, this was placed on its own page at the start of the survey and centered. More information on this question can be found here: https://www.qualtrics.com/support/survey‐platform/survey‐module/editing‐questions/question‐types‐guide/advanced/captcha‐verification/. *Our response to fraudulent sign‐ups*: We added a second CAPTCHA verification between screening and the first‐round survey.	T0, T1, T2, T3	Prevention, mitigation
c.	*Qualtrics* in‐built survey features (fraud detection elements)	The following fraud detection elements were enabled in *Qualtrics*: *Prevent multiple submissions*: This identified duplicates or respondents from the same IP address or location. For example, if true (1): the response is likely a duplicate (i.e., the participant has likely enrolled more than once). *Bot detection*: This is a form of automated bot detection that flagged likely bots with an invisible reCAPTCHA. For example, a score of < 0.5 out of 1: the respondent is likely a bot. *Security scan monitor*: This prevented security scanners from accidentally starting the survey (e.g., if a participant's computer has a security scanner that checks for safe links/websites before letting them in). *ReleventID*: This used the respondent's metadata (e.g., browser, operating system, location) to determine the likely fraud score and flag potential fraudsters. For example, a score of ≥ 75 out of 100: the response is likely a duplicate. *Qualtrics* has since replaced this feature with Duplicate detection. We opted to flag responses (i.e., allow the participant to complete the remainder of the survey but record fraud score for later review) instead of terminating the survey immediately, as we anticipated participants using the same network or device (e.g., organization staff or clinicians using the same WiFi network such as a VPN or the same workplace computer, lived experience participants in the same household). Fraud scores were provided by *Qualtrics* and manually reviewed by the research team. For example: a score of ≥ 30 out of 130: the response is likely fraudulent or a bot. More information, including detail on how to interpret fraud scores, can be found here: https://www.qualtrics.com/support/survey‐platform/survey‐module/survey‐checker/fraud‐detection/. Since utilizing these fraud detection elements, *Qualtrics* has changed the available features, where *RevelentID* has been replaced with *Duplicate Detection*.	T0/T1, T2, T3	Detection
d.	IP address review	IP address (e.g., 129.96.87.31) was automatically recorded by *Qualtrics* and included in the data. We conducted a manual review of IP addresses using a publicly available website to check its location and hosting server. For example, does this match participant's self‐reported location/postcode, is this within the expected enrolment geographic location (e.g., Australia‐only participants were in Australia).	T0/T1, T2, T3	Detection
e.	Unique (personalized, single‐user) survey links	For subsequent rounds of data collection (i.e., second‐ and third‐round surveys), unique, single‐user survey links were emailed directly to participants using the contact information they provided in the first‐round survey. By doing this we were able to send unique survey links to participants to prevent links from being shared or used multiple times.	T2, T3	Prevention
f.	Attention (validity) check questions	Three attention check questions, presented in the same format as other survey questions, were utilized in the first‐ and second‐round surveys and item order was randomized so these appeared at random points during the survey. A decision was made to not include attention check questions in the third‐round survey due to its brevity. For example, “For this item, please select the response option ‘Should not be included’ for both early intervention and augmenting treatment.” The research team manually reviewed participant responses against correct responses, and each response was scored as valid/not valid, yielding an overall validity score ranging from 0 (*no valid responses*) to 3 (*3 valid responses*). Scores of < 2 were flagged as *likely fraudulent*. *Our response to fraudulent sign‐ups*: We modified all attention check questions in the first‐round survey to require new correct responses. For example, where the previous correct response was ‘Should not be included’, the new correct response became “Essential.”	T1, T2	Detection, mitigation
g.	Open‐ended questions	Several open‐ended questions were utilized at each time point. For example, participants were asked to provide their name and residential postcode in a free‐text format with no validation. The research team manually reviewed participant responses to look for nonsensical, contradictory, identical responses.	T0, T1, T2, T3	Detection
h.	Cross‐referenced or duplicate questions	At each time point, participants were asked to provide their name and residential postcode. The research team manually reviewed participant responses to check for inconsistencies across time points. *Our response to fraudulent sign‐ups*: We added a duplicate age item during the first‐round demographics questions to check for consistency with age reported in screening, which was reviewed by the research team.	T0, T1, T2, T3	Detection, mitigation
i.	Multiple data points	Several data points were included in the study design (i.e., screening survey and three Delphi survey rounds). Further, completion of all three Delphi rounds was required to for reimbursement.	T0, T1, T2, T3	Prevention
j.	Affirming non‐fraudulent participation	The following was included in the consent statement: “I am not a fraudulent, fake, or imposter participant or a bot.”	T0	Prevention
k.	Targeted recruitment	Targeted recruitment of known researchers specializing in EDs occurred via group email.	—	Prevention
l.	Careful consideration of reimbursement (type, conditions, timing)	Participants received digital gift cards only valid in Australia or their country of residence (for international participants) only after completing all surveys, and compensation was not automated, allowing the research team time to screen the data beforehand.	—	Prevention, detection
m.	Transparency around fraudulent participation and eligibility for reimbursement	The following was included in the participant information sheet: “We acknowledge that there has been an increase in fraudulent participation in online research which can have a significant negative impact on research findings. Please note that participants will only be compensated once and participants will not be compensated if they are found by the research team to have submitted duplicate, ineligible, or fraudulent entries.”	T0	Prevention
n.	Satisficing versus fraudulent responses	To differentiate satisficing (i.e., providing quick, “good enough” answers rather than optimal ones; see Hamby and Taylor 2016 for overview) from fraudulent responses and reduce the risk of incorrectly identifying genuine participants as fraudulent, multiple sources of information (a combination of ‘red flags’) were used to identify fraudulent participants, not just one or two.	T0/T1, T2, T3	Detection
o.	Fraudulent participants' profile (a list of “red flags”; see Table 2)	We developed a profile (a list of “red flags”; see Table 2) of what we expected a fraudulent participant to look like, such as suspicious or unlikely personal information such as name, email address, phone number, similar response patterns among participants signing up within a short timeframe, speed and time of survey completion, inconsistency in information overtime. This list was initially drawn from existing literature (e.g., Davies et al. 2023; Lawlor et al. 2021; Roehl and Harland 2022; Storozuk et al. 2020; Teitcher et al. 2015) but was further refined following an analysis of the characteristics and response patterns observed among participants in the initial surge who were identified as *likely fraudulent*. It was then continuously updated throughout the study, to reflect the changing nature of fraudulent activity. The research team manually reviewed participants against this list at each time point and participants were given an overall fraudulent status of genuine participant, or suspected or likely fraudulent participant (see Figure 2).	T0/T1, T2, T3	Detection, mitigation
p.	Data quality checks	The following data were reviewed by the research team for each new sign‐up (postround one) and subsequent survey completion (postrounds two and three): full name, residential postcode, survey metadata (e.g., IP address, IP address location, user language, time of completion), *Qualtrics* fraud detection indicators (e.g., reCAPTCHA score, *RelevantID* duplicate, duplicate score, and last start date, fraud score), responses to attention check questions, and overall validity score (i.e., number of correct responses). For new sign‐ups only, additional checks included: valid email format and a valid mobile phone number based on country of residence (for Australian and International participants). For each subsequent survey (rounds two and three), the following were checked for consistency with baseline (round one) data: full name, residential postcode, and IP address location.	T0/T1, T2, T3	Detection

Abbreviations: CAPTCHA = revised completely automated public turing test to tell computers and humans apart; ED = eating disorder; IP = Internet protocol; reCAPTCHA = revised CAPTCHA; T0 = screening survey; T1 = first‐round survey; T2 = second‐round survey; T3 = third‐round survey; VPN = virtual private network.

^a^ References supporting the use of each strategy is presented in Table 3.

^b^ As the first‐round survey (T1) followed immediately after the screening survey (T0), some strategies were implemented at the T0/T1 timepoint.

^c^ We report the category utilized in the current study; however, other categories may apply based on how the strategy is applied.

### 2.3 The Surge of Fraudulent Participants

**FIGURE 1:**
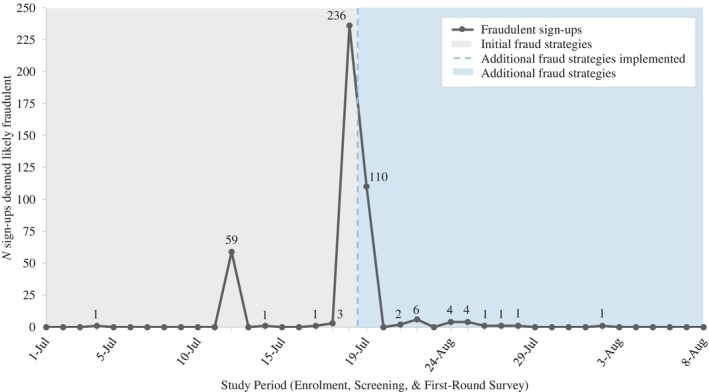
Daily fraudulent participants through enrolment, screening, and the first round survey between July 1, 2024 and August 8, 2024. Dotted line in indicates the point at which the research team implemented additional fraud strategies during this phase of the study (July 19, 2024, 1.53 p.m. ACST). Data labels provided for *n*'s > 0.

Despite the fraud strategies implemented, we were inundated with fraudulent sign‐ups (*n* = 431) over the first round of the Delphi study, between July 4, 2024 and August 2, 2024, as shown in Figure 1. The first suspicious sign‐up (*n* = 1) was detected 3 days after the study's launch. One week later there was a sudden surge in sign‐ups (73 in 1 day vs. ~11 per day) associated with multiple “red flags” identified during manual data checks. These red flags showed a similar pattern: a *Qualtrics* fraud score > 0; failed two or all three attention checks; identical start/completion times (e.g., clusters of sign‐ups starting at 2:06 p.m.); invalid Australian mobile numbers (e.g., incorrect format or landline); IP address from another country; invalid or mismatched postcodes (e.g., did not match postcode reported in the screening survey); and suspicious email addresses, such as those unrelated to the participant's name (e.g., sophieblogg96@gmail.com used by someone signed up as Melanie A. Rowley), containing random strings or numbers (e.g., s7035240593@gmail.com), using a similar format (e.g., first name and last name followed by numbers, michaelava9999@gmail.com), or repeatedly using the same email provider (e.g., clusters of sign‐ups using @outlook.com addresses). Some of these sign‐ups also occurred at unlikely hours for their reported timezone (e.g., between 9:00 p.m. and 5:00 a.m.).

One week after this surge in fraudulent sign‐ups, the number of fraudulent participants had risen to 301, suggesting that our existing fraud prevention strategies were no longer effective. We implemented new fraud strategies (specified below) that same day. While a further 110 fraudulent sign‐ups occurred on the same day the measures took effect, we believe these sign‐ups had already been initiated prior to the implementation of the new strategies. Only 20 additional cases were detected over the subsequent two weeks, occurring sporadically before declining to zero, and no further cases were detected after August 2, 2024. An additional two fraudulent participants were detected during the second‐round survey, on August 15 and 28, 2024, and no cases were detected during the third‐round survey. Although this cessation of fraudulent activity could suggest that the fraud prevention strategies implemented in subsequent rounds were working as intended, this was not tested empirically or statistically. Other explanations are also possible, such as a decline in automated bot activity or shifts in the focus of fraudulent participation (e.g., migration to other platforms, higher‐incentive studies). Notably, 22 fraudulent participants were still detected after the introduction of additional fraud strategies, highlighting that these measures did not fully deter or prevent all fraudulent sign‐ups. Fraudulent participants, alongside ongoing technological advances, are rapidly developing more sophisticated ways to circumvent safety measures (e.g., Dupuis et al. 2019; Simone et al. 2024; Ye et al. 2018), indicating that no strategy is foolproof (Walker et al. 2026).

### 2.4 Response to Surge of Fraudulent Participants

We thoroughly examined our internal procedures and the fraudulent sign‐ups during this period to identify possible causes of the surge. We discovered that this surge coincided with a public X/Twitter post by an ED organization, which shared the study hyperlink. The post was viewed 212 times and reposted by one of the group's 34.8k followers. It became clear that some fraudulent participants may have identified our eligibility criteria and used this knowledge to pass the screening survey and gain access to the study. In addition, some appeared to bypass other safeguards, such as the reCAPTCHA verification, postcode validation (e.g., AU postcode for Australian participants), and attention checks, by providing plausible responses to appear as legitimate participants. Bots can increasingly be programmed to bypass security measures such as CAPTCHA (e.g., Dupuis et al. 2019; Simone et al. 2024; Ye et al. 2018) and produce high‐quality, coherent survey responses resembling human reasoning (Walker et al. 2026; Westwood 2025).

We then implemented additional fraud mitigation strategies including: (a) adding a second reCAPTCHA between screening and the first‐round survey; (b) adding a duplicate age item in the first‐round demographic questions to check for consistency with screening responses; and (c) modifying attention check questions to require new correct responses (see Table 1 and Figure 1). Several other evidence‐informed measures were considered, such as replacing the original survey link, using unique links for the screening and first‐round surveys, requiring phone or video verification, and issuing reimbursement via postal mail instead of email, but we felt these were not feasible. Replacing the original link would have required recalling advertisements already widely distributed by ED organizations, risking confusion. Unique links would introduce additional steps for the participant that might deter participation for an already hard‐to‐reach population. Phone or video verification was not used because it was inconsistent with the study's online survey design, could undermine anonymity and honest responding given the sensitive topic, may deter participants with appearance‐related concerns who do not want to appear on camera, and would not have been feasible for our planned large‐scale recruitment and international sample. Lastly, postal mail reimbursement was not feasible due to cost, budget constraints, and privacy concerns.

### 2.5 Managing Suspected or Likely Fraudulent Participants

We outline a three‐step procedure for identifying and managing suspected or likely fraudulent participants, adapted from Davies et al. (2023). Whilst critical in ensuring valid data, we note it is time consuming for the research team and may inadvertently deter genuine participants, particularly, within a population that is already difficult to engage in research.

**FIGURE 2:**
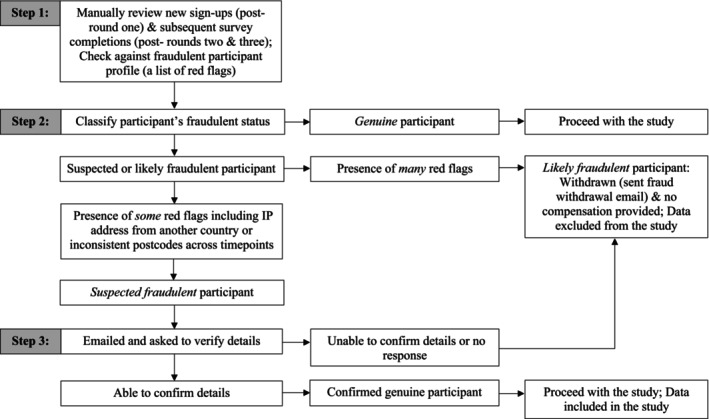
Flowchart for identifying and withdrawing suspected or likely fraudulent participants. Figure adapted from Davies et al. (2023).

First, we developed a procedural checklist for managing suspected or likely fraudulent participants; see Table 1 for the full list, as these had not yet been manualized. The protocol is summarized in Figure 2, which presents a flowchart detailing the steps taken to identify and withdraw suspected or likely fraudulent participants. First, two members of the research team manually reviewed each new sign‐up (post‐round one) and subsequent survey completion (postrounds two and three). This involved checking a comprehensive set of data (e.g., participant details, demographics, survey responses) against a fraudulent participants' profile, which outlined expected “red flags” for identifying likely fraudulent participants (Table 2). This list of red flags was initially informed by previous research identifying fraudulent responses (e.g., Davies et al. 2023; Lawlor et al. 2021; Roehl and Harland 2022; Storozuk et al. 2020; Teitcher et al. 2015) and further refined through analysis of the characteristics and patterns observed in the initial surge of likely fraudulent participants. It was then iteratively updated throughout the study as new fraudulent cases emerged to ensure it remained relevant and responsive to evolving indicators of fraud.

**TABLE 2:** Fraudulent participants' profile (a list of “red flags”).

Data category	Variable/other	Participant characteristics or response patterns with examples
Personal information	Full name	Suspicious or unlikely names, for example: Duplicates or similar names (e.g., repeated last name like Bentley, first and last name swapped, or exact duplicates such as Eli Flynn appearing twice) Unusual names (e.g., Adair Childe, Rex Wine, Mohammed Bacon, Creed Self) or names with irregular or unlikely spelling (e.g., Yehudith, Christeena, Pag) Names of famous people or celebrities (e.g., Tony Garcia, George Mason, Marsha Mason) First or last name only (e.g., Marcy)
	Email address	Suspicious email addresses, for example: Emails unrelated to the participant's name or associated with a different name (e.g., admireerror@gmail.com, sophieblogg96@gmail.com used by someone signed up as Melanie A. Rowley) Emails containing random strings or numbers (e.g., mrhwvrgmlcm@gmail.com, s7035240593@gmail.com) Emails followed a similar format (e.g., different iterations of first name and last name followed by numbers, such as apearce9@gmail.com, nigelb784@gmail.com, michaelava9999@gmail.com) Same email provider (e.g., clusters of sign‐ups using@outlook.com addresses) Keep in mind that these may be legitimate email addresses for some genuine participants.
	Mobile phone number	Invalid mobile phone number based on country of residence (for Australian and International participants), for example: Incorrect format (e.g., too few or too many digits, irregular spacing, phone numbers beginning with +, 61, or 4, as expected for Australian participants) Landline provided instead No phone number provided While not all of these mobile phone numbers are technically invalid, we observed that genuine participants typically entered numbers in the standard Australian format (i.e., beginning with 04 and spaced as 04XX XXX XXX), whereas fraudulent participants frequently used inconsistent or non‐standard formats.
Demographics	Stakeholder panel/group	Suspicious panels/groups reported, for example: Similar panels types (e.g., consecutive sign‐ups identifying as lived experience or significant others) Sudden influx of a specific panel without a corresponding change in recruitment of that group (e.g., a spike in sign‐ups identifying as clinicians)
	Residential postcode	Invalid postcode based on country of residence (for Australian and International participants), for example: Incorrect format (e.g., too few or too many digits) Full, invalid address entered in postcode Nonsensical entry or information other than postcode (e.g., age reported instead) Similar or unlikely postcodes (e.g., clusters of sign‐ups with the same postcode, or central business district postcodes) No postcode provided Inconsistent postcode reporting across timepoints (e.g., postcode for round one did not match postcode reported in the screening survey)
	Age	Suspicious age reported, for example: Age was not within the inclusion range of 18 or older Irregular format (e.g., age of adults reported in months) Age patterns (e.g., consecutive sign‐ups with ages increasing by 2‐year intervals or clustered within a narrow range) Nonsensical entry or information other than age Age not provided Inconsistent age reporting (e.g., age provided in round one did not match age reported in the screening survey, completed at the same timepoint)
Survey metadata	IP address and location and hosting server	Suspicious IP addresses, for example: IP address from another country (Australian participants) or didn't match the country of residence (International participants) Duplicate IP address (e.g., more than one sign‐up with the same IP address) IP address is not within the expected enrolment geographic location Keep in mind that multiple genuine participants can have the same IP address (e.g., participants using the same network or device such as organization staff or clinicians on a workplace VPN or computer, or lived experience participants from the same household).
	User language	Language other than EN = English
	Start or completion time	Suspicious start or completion times, for example: Survey completed at unlikely times of day (e.g., between 9:00 p.m. and 5:00 a.m.) Identical start or completion times (e.g., clusters of sign‐ups starting at 2:06 p.m., or completing at 2:53 p.m.)
	Duration	Survey completed significantly faster than expected (e.g., round one < 15 min, round two < 8–10 min, round three < 2 min), representing approximately half the estimated completion time
*Qualtrics* in‐built survey features (fraud detection elements)	ReCAPTCHA score	A score of < 0.5 out of 1 (e.g., 0.2, 0.4)
	Relevant ID duplicate	A score of TRUE (1)
	Relevant ID duplicate score	A score of ≥ 75 out of 100 (e.g., 90, 100)
	Relevant ID last start date	Recorded last survey date reported
	Fraud score	A score of ≥ 30 out of 130 (e.g., 45, 90, 105)
Failed attention checks	Attention (validity) check questions and overall validity score	Failed two or all three attention check items (e.g., validity score of 0 [*no valid responses*] or 1 [*1 valid response*])
Response patterns within a single survey	Delphi statements	Suspicious response patterns, for example: All items were rated as “Important” or “Essential,” reflecting the highest ratings All items were rated the same for early intervention and for augmenting treatment
Other	Sign‐ups	Influx/large number of sign‐ups in a short amount of time (e.g., > 20 sign‐ups in a day, or in noticeable intervals, such as every 3–20 min for 3–6 h block; e.g., Davies et al. 2023), particularly if no change or increase in recruitment efforts (e.g., no active advertising or outreach).
	Emails to the researcher	Suspicious emails to the researcher, for example: Asking about reimbursement before study completion Emails with identical or similar language Emails containing unusual phrasing or inconsistent English grammar or spelling

*Note:* This table presents a list of red flags—developed from previous research and analysis of response patterns among participants deemed likely fraudulent—outlining characteristics and response patterns expected of likely fraudulent participants, with examples. This list is not exhaustive, but these are some of the main fraud indicators. While the exact combination of red flags varied, multiple indicators were used to assess fraudulent status. Individual red flags were noted, as each contributed to the overall review; however, one or two alone were not considered enough to raise suspicion, since genuine participants may trigger these by mistake (e.g., a typo on one occasion or a legitimate email address like joebloggs246@gmail.com). In contrast, suspected or likely fraudulent participants often triggered numerous red flags, giving an overall impression that something is not right. Table and note adapted from Davies et al. (2023).

Abbreviations: IP = Internet protocol; reCAPTCHA = revised completely automated public turing test to tell computers and humans apart; VPN = virtual private network.

Second, two researchers independently classified participants into three categories, informed by Davies et al. (2023), though the category labels and criteria differed. First, *likely fraudulent* participants whose data displayed *many* red flags listed in Table 2. Simone et al. (2024) recommends a threshold of three or more infractions before withdrawal is considered. Second, *suspected fraudulent* participants with *some* red flags which included either an IP address from another country or inconsistent postcodes across timepoints, but were able to confirm their details when contacted and whose data were overall logical and consistent. Third, *genuine* participants with minimal red flags (typically one or two), whose responses were overall credible, non‐contradictory, and consistent across timepoints. Any unclear cases were resolved by discussion. While participants categorized as genuine were able to proceed with the study as normal, those that were categorized as likely fraudulent were sent a “fraud withdrawal email” informing them of their immediate withdrawal from the study due to suspected fraudulent activity. The email also stated that no compensation would be provided and invited them to contact the research team if they believed the decision was made in error. Given our transparency around fraudulent participation and eligibility for reimbursement in the participant information sheet prior to consent (listed in Table 1), we felt that withdrawing these participants and not providing compensation was ethically appropriate.

Third, participants that were categorized as suspected fraudulent were emailed and asked to verify their current residential address and postcode via self‐report in order continue with the study and be eligible for reimbursement. Those who were able to verify their details within the expected geographic location (e.g., within Australia for Australian participants) were categorized as genuine participants and given the opportunity to continue in the study. Those who failed to confirm these details or did not respond were classified as likely fraudulent and immediately withdrawn. Data from participants categorized as likely fraudulent were excluded from the study, whereas data from suspected fraudulent participants who were able to successfully verify their information were included.

### 2.6 Persons With Lived Experience Statement

Persons with lived experience participated in this study, and their deidentified data support the findings, however, they did not have a role in the study's conceptualization, design, data collection, analysis, interpretation, manuscript preparation, or decision to submit the paper for publication.

## 3 Practical Recommendations

Based on our experience with fraudulent participants, existing literature across multiple research areas, and emerging studies in EDs, we have collated 52 straightforward, practical evidence‐informed recommendations to help future researchers more effectively prevent, detect, and manage fraudulent participants in online research, summarized in Table 3. For a detailed list of specific features, real‐world examples, and additional considerations for each of these recommendations see Supporting Information S1.

**TABLE 3:** Practical recommendations for preventing, detecting, and managing fraudulent participants in online research.

Aspect of the study	Recommendation^a^	Category^b^	References
Study procedure and design	Use as many fraud prevention and detection strategies as possible.	Prevention, detection, mitigation	Davies et al. (2023); Godinho et al. (2020); Lawlor et al. (2021); Storozuk et al. (2020)
	Use multiple data points where possible.	Prevention, detection	Kramer et al. (2014)
	Include a real‐time or live questioning component during the study (e.g., identity verification during screening or enrolment).	Prevention, detection	Roehl and Harland (2022); Saberi (2020); Teitcher et al. (2015)
	Develop a plan to recontact suspected or likely fraudulent (suspiscious) respondents. See Figure 2 for an example of this procedure.	Detection, mitigation	Davies et al. (2023); Kramer et al. (2014)
	Incorporate PPI to help balance participants' needs with use of fraud strategies.	—	Davies et al. 2023
	Create a fraudulent profile (a list of red flags). See Table 2 for an example.	Prevention, detection	Davies et al. (2023); Teitcher et al. (2015)
	Differentiate satisficing versus fraudulent responses. See Supporting Information S1.1 for full description.	Detection	Hamby and Taylor (2016)
Informed consent	Be transparent about fraudulent participation.	Prevention	Teitcher et al. (2015)
	Have participants affirm nonfraudulent participation during consent.	Prevention	—
	Be transparent about the identifiable information collected and its use for study eligibility and fraud detection.	Prevention	Roehl and Harland (2022)
Screening	Use a presurvey screener or screening questions.	Prevention, detection	Lawlor et al. (2021); Pozzar et al. (2020); Roehl and Harland (2022)
	Ask potential participants to report how and where they heard about the research study and provide evidence of this.	Prevention, detection	Kramer et al. (2014); Nosek et al. (2002); Pozzar et al. (2020); Teitcher et al. (2015)
	Collect contact information and authenticate manually before enrolment.	Prevention, detection	Godinho et al. (2020); Lawlor et al. (2021); Roehl and Harland (2022); Teitcher et al. (2015)
Technical and software security measures	Enable in‐built fraud detection features in data collection platforms used for open recruitment (e.g., *Qualtrics*, *RedCap, LimeSurvey, SurveyMonkey*) and manually review embedded data (e.g., fraud scores). See Table 1 for further information on *Qualtrics* fraud detection features.	Prevention, detection, mitigation	Godinho et al. (2020); Teitcher et al. (2015)
	Use automated bot detection such as CAPTCHA or reCAPTCHA verification and manually review the associated embedded data.	Prevention, detection, mitigation	Davies et al. (2023); Dupuis et al. (2019); Godinho et al. (2020); Kramer et al. (2014); Lawlor et al. (2021); Pozzar et al. (2020); Storozuk et al. (2020); Teitcher et al. (2015); Walker et al. (2026)
	Consider blocking the use of VPNs.	Prevention, detection	Davies et al. (2023)
	Enable cookies.	Prevention, detection	Godinho et al. (2020); Lawlor et al. (2021); Teitcher et al. (2015)
Survey design	Include attention check questions (also called validity questions or trap questions) and manually review responses to check for validity/correctness.	Detection, mitigation	Davies et al. (2023); Lawlor et al. (2021); Liu and Wronski (2018); Storozuk et al. (2020)
	Include open‐ended or free‐text questions without validation and manually review responses.	Detection	Davies et al. (2023); Perkel (2020); Pozzar et al. (2020); Simone et al. (2024); Storozuk et al. (2020)
	Include cross‐referenced or duplicate questions and manually review responses for inconsistencies.	Detection, mitigation	Davies et al. (2023); Kramer et al. (2014); Nosek et al. (2002); Teitcher et al. (2015); Walker et al. (2026)
	Include honey‐pot questions (also called hidden items) such as using JavaScript.	Detection	Davies et al. (2023); Perkel (2020); Pozzar et al. (2020); Storozuk et al. (2020)
	Include reverse scored items.	Detection	Johnson (2005); Storozuk et al. (2020)
	Include illogical options on multiple choice questions.	Detection	Davies et al. (2023); Nosek et al. (2002); Storozuk et al. (2020)
	Do not include a back or previous button.	Prevention	Teitcher et al. (2015)
	Randomize or change the order of questions/question blocks where possible.	Prevention, detection	Teitcher et al. (2015)
	Present text or instructions as an image or distorted image.	Prevention, detection	Storozuk et al. (2020)
Financial incentives and compensation	Carefully consider financial incentives.	Prevention	Davies et al. (2023); Storozuk et al. (2020); Teitcher et al. (2015); Wright (2005)
	Consider lowering incentives and/or emphasizing the importance of research.	Prevention	Storozuk et al. (2020); Teitcher et al. (2015); Wright (2005)
	Consider conducting a lottery rather than providing individual payments.	Prevention	Kramer et al. (2014); Teitcher et al. (2015); Wright (2005)
	Consider providing non‐financial incentives.	Prevention	Storozuk et al. (2020); Wright (2005)
	Consider using incentives only valid in the host country.	Prevention, detection	Davies et al. (2023)
	Do not automate compensation.	Prevention, Detection	Konstan et al. (2005)
	Consider sending reimbursement in the post/mail (vs. email).	Prevention, detection	Davies et al. (2023); Teitcher et al. (2015)
	Consider providing reimbursement contingent on completion of all timepoints or break down reimbursement into smaller chunks for each timepoint.	Prevention	Teitcher et al. (2015)
	Be clear regarding eligibility for compensation.	Prevention	Davies et al. (2023); Teitcher et al. (2015)
	Consider not publicly advertising the amount or type of compensation that will be provided.	Prevention	Davies et al. (2023); Kramer et al. (2014); Lawlor et al. (2021)
Survey distribution and recruitment	Use targeted survey distribution and recruitment outside of social media where possible.	Prevention	Jones et al. (2021); Lawlor et al. (2021); Nosek et al. (2002); Storozuk et al. (2020)
	Do not publicly disclose the study eligibility criteria.	Prevention	Lawlor et al. (2021); Pequegnat et al. (2007); Pozzar et al. (2020)
	Use unique, single‐user survey links.	Prevention, detection	Godinho et al. (2020); Jones et al. (2021); Lawlor et al. (2021); Pozzar et al. (2020); Storozuk et al. (2020); Teitcher et al. (2015)
	Restrict survey access with invitation, access code, or password.	Prevention	Pozzar et al. (2020)
	Shut down the survey link.	Mitigation	Storozuk et al. (2020)
	Do not share the study link publicly.	Prevention	Perkel (2020); Pozzar et al. (2020); Storozuk et al. (2020); Teitcher et al. (2015)
	Track or search for the survey URL online.	Prevention, detection, mitigation	Teitcher et al. (2015)
Data quality checks	Regularly check incoming data.	Detection	Davies et al. (2023); Godinho et al. (2020); Konstan et al. (2005); Lawlor et al. (2021); Perkel (2020); Pozzar et al. (2020); Storozuk et al. (2020); Teitcher et al. (2015)
	Manually check for inconsistencies or illogical data, appropriate to the study.	Detection	Davies et al. (2023); Kramer et al. (2014); Perkel (2020); Pozzar et al. (2020); Roehl and Harland (2022); Teitcher et al. (2015)
	Manually check for response patterns.	Detection	Davies et al. (2023); Lawlor et al. (2021); Perkel (2020); Storozuk et al. (2020); Teitcher et al. (2015)
Survey metadata	Collect and manually review survey metadata.	Detection	Godinho et al. (2020); Konstan et al. (2005); Lawlor et al. (2021); Pozzar et al. (2020); Storozuk et al. (2020); Teitcher et al. (2015)
	Manually review time of survey completion.	Detection	Davies et al. 2023; Konstan et al. (2005); Kramer et al. (2014); Lawlor et al. (2021); Pozzar et al. (2020); Storozuk et al. (2020); Teitcher et al. (2015)
	Manually review survey response time (or speed of survey completion).	Detection	Lawlor et al. (2021); Pequegnat et al. (2007); Storozuk et al. (2020); Teitcher et al. (2015); Walker et al. (2026)
	Manually review geolocation and IP address.	Detection	Godinho et al. (2020); Lawlor et al. (2021); Roehl and Harland (2022); Storozuk et al. (2020); Teitcher et al. (2015)
	Check user language (survey embedded data).	Detection	—

Abbreviations: CAPTCHA = revised completely automated public turing test to tell computers and humans apart; ED = eating disorder; IP = Internet protocol; PPI = patient and public involvement; reCAPTCHA = revised CAPTCHA; VPN = virtual private network.

^a^ See Supporting Information S1 for extended list of practical recommendations with specific features, examples, and additional considerations.

^b^ Several categories may apply to a single strategy based on how it is applied.

This list is not exhaustive, nor are we the first or only research team to propose such recommendations in ED and other research areas (see Bonnamy et al. 2025; Comachio et al. 2025; Davies et al. 2023; Lawlor et al. 2021; Ménard et al. 2025; Roehl and Harland 2022; Simone et al. 2024; Storozuk et al. 2020; Teitcher et al. 2015; Yarrish et al. 2019), this is, to our knowledge, the largest set of practical, easy‐to‐implement recommendations in ED research. Davies et al. (2023) was especially influential in guiding strategy selection and use, offering detailed evidence for 11 broad (15 specific) practical recommendations to prevent or detect fraudulent responses across key areas. We build on and integrate this existing work by compiling a wide range of evidence‐informed strategies from across research areas and presenting them in a clear, accessible table to support researchers in applying them more easily. We further extend this work by providing detailed practical guidance and examples that serve as a how‐to resource for researchers.

Although these recommendations were developed in the context of ED research, they also draw on emerging literature from other fields and are therefore applicable across research domains. While some strategies are based on our experience using *Qualtrics*, these are likely applicable to similar survey platforms used for open recruitment (e.g., *RedCap*, *LimeSurvey*, *SurveyMonkey*). We note that the risk of fraud and mitigation approaches can vary across platforms. For example, crowd‐sourcing and panel‐based platforms (e.g., *MTurk*, *Prolific*, *Qualtrics Panels*) typically include in‐built, layered safeguards, such as account verification, eligibility screening, quality tracking, and location/device checks, combined with researcher review, which can reduce fraud risk and partly offset the need for additional researchers‐implemented strategies.

We acknowledge that many recommended fraud strategies are resource‐intensive, placing a considerable burden on researchers and participants. For researchers, these demands may increase workload, staffing needs, and study costs, while for participants, additional screening measures, attention checks, or verification procedures may increase the time commitment and survey burden. Researchers should carefully consider which strategies are appropriate and feasible, balancing the need to safeguard data quality against practical, ethical, and participant‐related burdens.

### 3.1 Additional Literature on Managing Fraudulent Participation

While the current research provides practical recommendations for detecting and managing fraudulent participants, three complementary areas of work fall outside its scope. First, an emerging literature advances statistical methods and indices for detecting fraudulent responses in survey data (Buchanan and Scofield 2018; Dupuis et al. 2019; Irish and Saba 2023; Pinzón et al. 2024). For example, Dupuis et al. (2019) conducted an empirical comparison of seven indices for detecting computer‐generated (i.e., nonhuman) responses in online questionnaires, using a large sample of real and simulated data, and determined the best estimators. Recently, Pinzón et al. (2024) systematically examined the performance of 31 fraud indicators, and combinations of these (i.e., indicator ensembles), using predictive power (i.e., a variation of precision that takes into account the proportion of fraud in the sample), and recall, and provide a thorough review of which methods were most effective (e.g., novel email address, consecutive submissions, improbable locations).

Second, several researchers have proposed frameworks to guide planning and study design decisions aimed at anticipating and mitigating survey fraud (e.g., Lawlor et al. 2021; Wessling et al. 2017). For example, Lawlor et al. (2021) developed the Reflect, Expect, Analyze, and Label (REAL) Framework, consisting of four sets of guiding questions, to guide researchers to identify and address suspected online survey fraud, particularly for those studies offering financial incentives where fraud risk is high.

Third, recent research has highlighted the capabilities of agentic AI models, such as the ChatGPT agent, to complete online surveys in ways that closely mimic human responses, bypassing once effective bot‐detection safeguards and threatening the validity of the data (Walker et al. 2026; Westwood 2025). In a Forum paper, Walker et al. (2026) describe the challenges researchers—often without AI expertise—face when screening for AI‐generated responses. They outline the most successful detection methods and provide practical recommendations, including testing screening strategies across multiple AI platforms. Researchers are encouraged to consult this broader literature alongside the recommendations provided in the current study to strengthen their overall approach to preventing and detecting fraud in online research.

## 4 Discussion

The growth of online research has been accompanied by increasing fraudulent participation and unusable data, undermining research integrity and posing a major threat to all online research studies. At the same time, continual advances in technologies such as AI have enabled the development of more sophisticated bots and automated software (e.g., systems using natural language processing and generative AI similar to ChatGPT and its agent model; Sager et al. 2021) that are capable of bypassing previously effective security measures and producing human‐like responses (Walker et al. 2026; Westwood 2025). It is essential for researchers to have an awareness and understanding of the current risks associated with online research and to be proactive in their implementation of antifraud practices from the outset, despite the increased burden to the research team. Researchers will need to remain attentive and responsive to the changing digital and research landscapes and stay informed with current antifraud practices (Donegan and Gillan 2022; Yarrish et al. 2019). In the absence of a gold standard, we strongly recommend that researchers employ a multifaceted, layered approach to fraud detection and management to safeguard online research, no one strategy alone is foolproof.

While we recognize that this approach is labor‐ and resource‐intensive and places a considerable burden on researchers and on participants, our current online randomized controlled trial, which incorporates several recommended strategies (e.g., a screening survey, manual review of sign‐ups, a fraudulent participant profile, attention check questions, avoiding publicly posting the survey link), suggests that these measures substantially reduce fraudulent sign‐ups from the outset. They have also enabled us to rapidly identify and remove likely fraudulent participants. Overall, we believe that implementing these strategies has significantly mitigated fraud and strengthened the integrity of our data.

### 4.1 Conclusion

Combining our experiences with insights from the existing literature and recent EDs studies addressing fraudulent participation, we highlight the inherent and growing risks faced by online research. We synthesize current evidence and provide simple, practical recommendations for preventing, detecting, and managing fraudulent participants, to assist future researchers in safeguarding online research against fraud. While grounded in ED research, our discussion draws on literature from multiple research fields, suggesting that these risks and recommendations are broadly applicable. Despite implementing a wide range of evidence‐informed strategies, we found that fraudulent participants continued to evade detection, even after additional safeguards were introduced. This underscores a harsh reality: as fraudulent participation becomes more sophisticated and widespread, and digital technologies advance rapidly, it is increasingly difficult for researchers to stay ahead. No single strategy can fully prevent fraud, and even once effective methods may soon become obsolete. We therefore urge researchers to implement multifaceted, evidence‐based strategies, stay vigilant, and continuously adapt to stay ahead of ever‐growing threats to online research.

## Author Contributions

**Jamie‐Lee Pennesi:** conceptualization, data curation, investigation, methodology, project administration, writing – original draft preparation, writing – review and editing. **Mia L. Pellizzer:** data curation, investigation, project administration, writing – review and editing. **Tracey D. Wade:** conceptualization, methodology, funding acquisition, resources, supervision, writing – original draft preparation, writing – review and editing.

## Lived Experience Involvement Statement

Persons with lived experience participanted in this study, and their deidentified data support the findings, however they did did not have a role the study’s conceptualisation, design, data collection, analysis, interpretation, manuscript preparation, or decision to submit the paper for publication.

## Funding

This work is funded by a National Health and Medical Research Council Investigator Grant (2025665) to Tracey Wade. Funding sources did not have a role in the study's conceptualisation, design, data collection, analysis, interpretation, manuscript preparation, or decision to submit the paper for publication.

## Disclosure

The views expressed in the submitted paper are solely the authors and do not necessarily represent the official views of the institution or funder.

## Conflicts of Interest

The authors declare no conflicts of interest.

## Supporting information


**Data S1:** eat70083‐sup‐0001‐supinfo.docx.
**Table S1:** Practical recommendations for preventing, detecting, and managing fraudulent participants in online research with specific features, examples, and additional considerations.

## Data Availability

The deidentified participant data that support the findings of this study are available on the Open Science Framework (OSF), https://osf.io/e4nbf/. Additional variables are available from the corresponding author upon reasonable request.
