# Supplementary material for: Safeguarding Online Research in Eating Disorders Against Fraud: Increasing Risks and Practical Recommendations
Source: Int J Eat Disord. 2026 Mar 31;59(7):1451–68. doi: 10.1002/eat.70083 (PMC13326784; doi:10.1002/eat.70083)
Supplement: Supplementary file 1 — Data S1: eat70083‐sup‐0001‐supinfo.docx. Table S1: Practical recommendations for preventing, detecting, and managing fraudulent participants in online research with specific features, examples, and additional considerations. [file EAT-59-1451-s001.docx]

*6^th^ February 2026*

**Supplemental Online Content**

**Safeguarding online research in eating disorders against fraud: Increasing risks and practical recommendations**

Jamie-Lee Pennesi, PhD^1^* ORCID: 0000-0003-1461-7303

Mia L Pellizzer, PhD^1^ ORCID: 0000-0002-9917-2069

Tracey D Wade, PhD^1^ ORCID: 0000-0003-4402-770X

^1^ Flinders University Institute for Mental Health and Wellbeing and Blackbird Initiative, Flinders University, South Australia, Australia

**Table of Contents**

| **Section** | **Title** | **Page** |
| --- | --- | --- |
| S1. | Supplementary Results | 3 |
| S1.1 | Extended Practical Recommendations  Table S1. Practical recommendations for preventing, detecting, and managing fraudulent participants in online research with specific features, examples and additional considerations. | 3 |
| S1.2 | References | 17 |

**S1. Supplementary Results**

**S1.1. Extended Practical Recommendations**

**Table S1.** Practical recommendations for preventing, detecting, and managing fraudulent participants in online research with specific features, examples and additional considerations.

|  | **Recommendation** | **Specific features, examples & additional considerations** |
| --- | --- | --- |
| *Study procedure & design* | | |
| 1. | Use as many fraud prevention & detection strategies as possible. | - Use multiple fraud prevention & detection strategies, rather than relying on one or two. Combining approaches increases accuracy in detecting fraudulent participants & reduces the likelihood that they will bypass security measures. |
|  |  | - Plan & apply these strategies from the outset & maintain them throughout the study. |
|  |  | - There is evidence that fraudulent participants are becoming more common & more sophisticated, so researchers should remain vigilant, stay informed with current antifraud practices, & be prepared to adapt strategies & methods as needed (Donegan & Gillan, 2022; Yarrish et al., 2019). |
| 2. | Use multiple data points where possible. | - This could deter fraudulent participants & increase the likelihood of catching them out (e.g., multiple timepoints may not be worth a fraudulent participants time, they may provide inconsistent/personal information over time). |
| 3. | Include a real-time or live questioning component during the study (e.g., identity verification during screening or enrolment). | - This may include an audio call, phone call, in-person visit, or video-platform call with the video on (e.g., Zoom, Teams). This could be a deterrent & another means to detect lying. |
|  |  | - Consider including this at screening or enrolment to confirm the participant is genuine before data collection. |
|  |  | - Keep in mind, this could deter fraudulent participation but may also discourage genuine participants (e.g., those who do not want to appear on camera), compromise anonymity and honest responding for sensitive topics, and may be impractical for large-scale recruitment. |
| 4. | Develop a plan to re-contact suspected or likely fraudulent (suspicious) respondents. | - Plan to re-contact respondents identified suspected or likely fraudulent (suspicious respondents) as part of the research protocol prior to data collection. For example, contact respondents with two or more red flags to confirm their contact or personal information. If respondents do not respond or cannot confirm their information this may be a sign of fraud. See **Figure 2** for an example of this procedure. |
| 5. | Incorporate PPI to help balance participants’ needs with use of fraud strategies. | - When developing fraud prevention & detection strategies, consider how to protect participants’ privacy while maintaining data quality (Davies et al., 2023; Roehl & Harland, 2022; Teitcher et al., 2015). |
|  |  | - Include PPI (e.g., consultation with key stakeholders such as people with lived experience) from the outset to ensure that participant needs are appropriately balanced & fraud strategies are acceptable & proportionate within the target population. For example, researchers may seek input on specific strategies, such as requesting contact information for verification when a response is flagged as potentially fraudulent. |
| 6. | Create a fraudulent profile (a list of red flags). | - Drawing on existing literature (e.g., Davies et al., 2023) develop a profile of indicators that may suggest fraudulent participation. See **Table 2** for an example. This may include patterns in personal information or survey responses that appear suspicious, invalid, inconsistent, illogical, or highly uniform. For example, unusual or repeated personal details (e.g., name, email address, phone number), identical or highly similar response patterns among participants signing up within a short period, unusually fast or oddly timed survey completion, or changes in personal information overtime. |
|  |  | - Regularly review & update these indicators based on any new patterns observed among confirmed fraudulent participants, & to reflect the changing nature of fraudulent activity. |
|  |  | - Share these indicators with other researchers & report them in any relevant research publications to support transparency & strengthen future fraud prevention efforts. |
| 7. | Differentiate satisficing versus fraudulent responses. | - Satisficing (i.e., providing quick, “good enough” answers rather than optimal ones; see Hamby & Taylor, 2016 for overview), can reflect inattentiveness, low effort, or misunderstanding and may cause genuine participants to be incorrectly identified as fraudulent. To reduce this risk, identify fraudulent participants using multiple sources of information (e.g., multiple red flags), rather than just one or two. |
|  |  | - A threshold of three or more infractions is recommended before withdrawal is considered (Simone et al., 2024). |
| *Informed consent* | | |
| 8. | Be transparent about fraudulent participation. | - In the participant information sheet, acknowledge awareness of fraudulent participation in online studies (e.g., “We acknowledge that there has been an increase in fraudulent participation in online research which can have a significant negative impact on research findings. As such, this study includes several fraud prevention & detection strategies.”). |
|  |  | - This signals to potential fraudulent participants that monitoring is in place, which may serve as a deterrent. |
| 9. | Have participants affirm non-fraudulent participation during consent. | - For example, include the following in the consent statement “The following was included in the consent statement: “I am not a fraudulent, fake, or imposter participant or a bot.” |
|  |  | - This also signals to potential fraudulent participants that monitoring is in place, which may serve as an additional deterrent. |
| 10. | Be transparent about the identifiable information collected & its use for study eligibility & fraud detection. | - In the participant information sheet, clearly state what identifiable information will be collected & what information will be used to determine eligibility & fraud status. |
|  |  | - Keep in mind, this may help deter fraudulent participants but could also dissuade genuine participants concerned about the collection of identifiable information. |
| *Screening* | | |
| 11. | Use a pre-survey screener or screening questions. | - Instead of using all yes/no or multiple choice questions, make one or two questions open-ended or free-text entry, with or without validation. For example, “Please provide your age in years*.*” [text-entry, number validation], “Please provide your residential postcode.” [text-entry, no validation]. |
|  |  | - Consider whether the screening questions lead straight into the subsequent survey or it is kept separate to allow you to manually pre-screen responses to check for potential fraud before data collection. The latter would allow you to use unique survey links for the subsequent survey which can prevent multiple fraudulent entries using the same link. |
| 12. | Ask potential participants to report how & where they heard about the research study & provide evidence of this. | - If feasible, ask participants to provide evidence of the recruitment source (e.g., links to webpages, posts, emails or other announcements about the study, email advertisement). If the information provided is suspicious, does not match recruitment efforts, or cannot be provided, this may indicate potential fraud. |
| 13. | Collect contact information & authenticate manually before enrolment. | - This could include verifying phone numbers via text or call, confirming email addresses, or verifying information through publicly accessible directories. This allows researchers to screen potential participants prior to enrolment & data collection. |
|  |  | - Researchers should consider what constitutes the minimal amount of information required to verify or confirm the discrepancy & avoid inquiring beyond this (see Jones et al., 2021). |
| *Technical & software security measures* | | |
| 14. | Enable in-built fraud detection features in data collection platforms used for open recruitment (e.g., *Qualtrics*, *RedCap, LimeSurvey, SurveyMonkey*) & manually review embedded data (e.g., fraud scores). | - Various fraud detection features exist in data collection platforms used for open recruitment (e.g., prevent multiple submissions, bot detection, duplicate responses; see **Table 1** for further information on *Qualtrics* features) depending on the survey platform used (e.g., *Qualtrics*, *RedCap,* *LimeSurvey*, *SurveyMonkey*), & the associated embedded data can be interpreted differently. |
|  |  | - Consider whether to *flag responses* (i.e., allow the participant to complete the survey but record fraud score for later review) or *terminate the survey* immediately. If you anticipate that multiple participants may use the same network or device (e.g., staff using the same WiFi network or same workplace computer, participants in the same household) it is recommended that you flag responses for later review, to avoid terminating the survey for legitimate participants. |
| 15. | Use automated bot detection such as CAPTCHA or reCAPTCHA verification & manually review the associated embedded data. | - Use this at least twice (e.g., at consent & start of the baseline survey); the second is an added layer of protection if a fraudulent participant gets through the first. Consider using it at least once at a later timepoint if conducting a longitudinal study. Also see Walker et al. (2026). |
|  |  | - Place CAPTCHA on its own page or with few questions, so that a participant doesn’t need to provide responses again if they need to redo the verification. |
|  |  | - Implement the latest version of CAPTCHA software to maximise security. |
|  |  | - There is evidence to suggest that some sophisticated bots can be programmed or trained to bypass security measures such as CAPTCHA (e.g., Dupuis et al., 2019; Simone et al., 2024; Ye et al., 2018), so do not rely solely on this strategy. |
| 16. | Consider blocking the use of VPNs. | - There are several IP filtering tools or third party services that can detect & flag or block traffic from known VPN/proxy/data centres, which can prevent location spoofing & reduce fraudulent traffic. |
|  |  | - Be mindful that legitimate participants may also use VPNs for privacy, institutional networks (e.g., a university WiFi network such as eduroam) or work devices. If you anticipate multiple participants using the same network or device, consider flagging VPN-linked responses for review instead, to avoid blocking legitimate participants. Consider asking a simple verification question if someone is flagged (e.g., postcode check, region-specific question). |
| 17. | Consider blocking foreign IP addresses. | - For example, if you are recruiting only Australian participants, consider blocking foreign/non-Australian IP addresses. |
|  |  | - This strategy is recommended instead of blocking sign-ups from duplicate IP addresses, as shared networks (e.g., workplaces, institutions, households) can result in legitimate participants appearing to have the same IP address (e.g., Davies et al., 2023). |
|  |  | - Be mindful that some genuine participants may be travelling or temporarily located overseas. Instead, consider flagging foreign IP addresses for review. Consider asking a simple verification question if someone is flagged (e.g., postcode check, country-specific question). |
|  |  | - Be mindful that use of a VPN can bypass IP address restrictions, so do not rely solely on this strategy. |
| 18. | Enable cookies. | - This allows researchers to detect repeat or suspicious survey submissions (e.g., multiple submissions from the same device) by tracking unique devices or sessions. |
|  |  | - Consider flagging suspicious responses for review instead of automatically excluding participants, as legitimate participants using the same device (e.g., workplace, institution, household) may be incorrectly flagged. |
|  |  | - Due to potential privacy concerns, the use of cookies should be explained in the participant information sheet & consent forms. |
|  |  | - Be mindful that fraudulent participants can clear cookies, use multiple devices, or switch browsers to bypass detection, so do not rely solely on this strategy. |
| *Survey design* | | |
| 19. | Include attention check questions (also called validity questions or trap questions) & manually review responses to check for validity/correctness. | - Include these a few times throughout (e.g., instructing participants to select a specific response option to check they are paying attention to the survey). For example, “Please select the response option ‘Never’ for this question”. Also consider using trick/bogus questions, requiring participants to respond to items that everyone should agree to (i.e., universal truths such as “I am human”) or disagree to (i.e., universal falsities such as “I can time travel”). |
|  |  | - Don’t just use one attention check question in case it’s a genuine mistake. Three attention check questions is ideal, with participants answering 2/3 or 3/3 questions correctly indicating a valid/correct response. |
|  |  | - Place attention check questions randomly throughout & randomise if possible so fraudulent participants can’t anticipate them or learn where they are. |
|  |  | - When selecting a specific response option, consider responses that are not typical responses in your sample population (e.g., low scores within a clinical sample where scores are typically high). Fraudulent participants who are not paying attention are likely to respond in a manner they feel is consistent with the sample population (e.g., providing all high scores to pretend they have an ED). |
|  |  | - There is evidence to suggest that some sophisticated bots can be programmed or trained to provide ‘correct’ responses, so consider changing the specific response option or attention check questions regularly & do not rely solely on this strategy. |
|  |  | - An instructional manipulation check can also be used to check participant attention (see Oppenheimer et al., 2009). |
| 20. | Include open-ended or free-text questions without validation & manually review responses. | - By not using validation, nonsensical, contradictory, unusual, or identical responses will be easier to spot (e.g., a child’s age not aligning with their reported year level). |
|  |  | - It is suggested that bots are more likely to provided nonsensical text or no response. |
|  |  | - Consider whether long format open-ended responses (e.g., 150-word response) may be necessary (see Walker et al., 2026). |
| 21. | Include cross-referenced or duplicate questions & manually review responses for inconsistencies. | - Ask the same question more than once (e.g., in the same or across different timepoints) or require the participant to respond in a different way (e.g., multiple choice vs. open-ended). For example, ask for demographic or identifying information that is unlikely to change over the study (e.g., date of birth, mobile number, email, residential postcode) at multiple points. If information is inconsistent (e.g., different residential postcode at screening & at baseline) this may indicate fraud. |
|  |  | - If information is inconsistent, consider emailing the participant to confirm the discrepancy without giving too much away. If they don’t respond or can’t reasonably verify or confirm the discrepancy (e.g., they provide a different postcode) this may be another sign of fraud. Consider removing them from study or flagging them as likely fraudulent. |
|  |  | - Be mindful that genuine participants can make mistakes by chance (e.g., they may have typed in their date of birth incorrectly on one occasion). |
|  |  | - If conducting a longitudinal study, be aware that depending on the length of time between assessments, some information may not stay consistent (e.g., weight, height, age). |
|  |  | - Researchers should consider what constitutes the minimal amount of information required to verify or confirm the discrepancy & avoid inquiring beyond this (see Jones et al., 2021). |
| 22. | Include honey-pot questions (also called hidden items) such as using JavaScript. | - Honey-pot questions are those which are hidden from human participants, but not from bots. These questions should resemble ‘real’ questions (e.g., “What is your marital status?”), but they are hidden from human participants. If a response is provided to this hidden question, this may indicate that the respondent is likely a bot. |
|  |  | - There is evidence to suggest that some sophisticated bots can detect & avoid honey-pot questions (Simone et al., 2023), so do not rely solely on this strategy. |
| 23. | Include reverse scored items. | - Including items that require reverse scoring where possible can help flag inconsistencies and patterned responding. For example, if a motivation scale contains both “I enjoy challenging tasks” and a reverse-scored item such as “I avoid difficult activities”, participants should respond in opposite directions. When they don’t, this could indicate inattention, insufficient effort, or automated responding. |
| 24. | Include illogical options on multiple choice questions. | - Include illogical options can be used to detect inattentive, careless, or automated responding. For example, a survey the question like “What is your gender?” could include obviously illogical options such as “I’m a unicorn” or “Purple elephant”. Genuine or careful participants are unlikely select these choices, but someone answering randomly or automatically might, which might indicate potential fraud. |
| 25. | Do not include a back or previous button. | - This makes fraud more difficult as fraudulent participants can’t easily copy responses & resubmit a survey. |
| 26. | Randomise or change the order of questions/question blocks where possible. | - This makes it difficult for fraudulent participants to anticipate questions, learn the order, or easily copy responses. |
| 27. | Present text or instructions as an image or distorted image. | - It is suggested that bots are incapable of decoding images of distorted text. |
|  |  | - There is evidence to suggest that some sophisticated bots can now decipher images/distorted images, so do not rely solely on this strategy. |
| *Financial incentives & compensation* | | |
| 28. | Carefully consider financial incentives. | - There is evidence to suggest that data validity issues are particularly common in studies offering financial incentives (Bowen et al., 2008; Konstan et al., 2005; Wright, 2005). |
|  |  | - If the study includes financial compensation, consider compensation approaches that minimise the risk of participant fraud (see examples below). |
| 29. | Consider lowering incentives &/or emphasising the importance of research. | - This will help to de-incentivise fraud & deter fraudulent participants from signing up for financial compensation. |
|  |  | - There is evidence to suggest that fraud is more prevalent when compensation is higher (Chandler & Paolacci, 2017). |
| 30. | Consider conducting a lottery rather than providing individual payments. | - Be mindful that this strategy may inadvertently increase multiple responses as participants try to increase the chances of winning. |
| 31. | Consider providing non-financial incentives. | - For example, consider providing incentives that are redeemable for real merchandise such as books or food instead. |
| 32. | Consider using incentives only valid in the host country. | - For example, if you are recruiting an Australia-only sample, provide vouchers only redeemable in Australia (e.g., Coles supermarket vouchers). |
|  |  | - Make this clear in the participant information sheet to deter fraudulent participants from signing up for financial compensation if they are in another country. |
| 33. | Do not automate compensation. | - Instead, delay sending reimbursement (e.g., a few days after study participation) to allow researchers time to review & detect fraudulent participants. If using email to send reimbursement, check for duplicate email addresses before sending. |
| 34. | Consider sending reimbursement in the post/mail (vs. email). | - Have the participant verify their residential address & manually authenticate before sending (e.g., is the address for a business or non-residential location?). |
|  |  | - Check if multiple vouchers are being sent to one location. |
| 35. | Consider providing reimbursement contingent on completion of all timepoints or break down reimbursement into smaller chunks for each timepoint. | - This could be a deterrent for fraudulent participants (e.g., prolonged study participation it is not worth their time). |
| 36. | Be clear regarding eligibility for compensation. | - To deter fraudulent participants from signing up for financial compensation, make it clear in the participant information sheet that participants will only be compensated once & will not be compensated if they are found by the research team to have submitted duplicate, ineligible, or fraudulent entries. |
|  |  | - This also signals to potential fraudulent participants that monitoring is in place, which may serve as an additional deterrent. |
| 37. | Consider not publicly advertising the amount or type of compensation that will be provided. | - Carefully consider how compensation is mentioned in public-facing advertisements, & instead, only mention compensation during informed consent (i.e., in the participant information & consent form). |
| *Survey distribution & recruitment* | | |
| 38. | Use targeted survey distribution & recruitment outside of social media where possible. | - For example, share study invitations to trusted groups (e.g., reputable ED organisations or research groups, lived experience communities) or send them directly to verified individuals (e.g., an existing database in your research team or department). This helps limit exposure to fraudulent participants. |
| 39. | Do not publicly disclose the study eligibility criteria. | - Consider limiting public disclosure of study eligibility criteria during recruitment & advertising. This will help to reduce the risk of fraudulent participants identifying correct responses to gain access to the study. |
|  |  | - Be mindful that this strategy may inadvertently deter some genuine eligible participants who do not recognise that they may qualify. |
| 40. | Use unique, single-user survey links. | - Make the study accessible only via unique (personal, single-user) survey links sent by the researcher. This allows the researcher to act as a ‘gatekeeper’, ensuring that only eligible participants who have been screened gain access to the study. |
|  |  | - Consider inviting potential participants to email the researcher & then complete a short eligibility screening before being sent the unique survey link, though this may reduce participant anonymity. It is important to collect only the information essential for determining eligibility. |
| 41. | Restrict survey access with invitation, access code, or password. | - Only permit access to individuals who have a personal invitation or require participants to enter an access code or password before beginning the study. |
| 42. | Shut down the survey link. | - Be prepared to immediately shut down the survey link as soon as signs of suspicious activity appear. |
|  |  | - Once fraudulent participants gain access to a survey link, fraudulent responses can escalate rapidly, potentially generating hundreds of unusable responses within hours. |
|  |  | - If the survey is compromised, the most effective action is to close the existing survey link & create a new, secure one. |
| 43. | Do not share the study link publicly. | - Instead, consider having interested participants contact the researchers to obtain the survey link. |
|  |  | - Be mindful that fraudulent participants may still email the researchers to obtain the study link, so do not rely solely on this strategy. |
|  |  | - There is evidence to suggest that fraudulent participants who intend to defraud research exchange information (e.g., the study link) about online research studies offering financial incentives, so consider using unique survey links. |
| 44. | Track or search for the survey URL online. | - This allows researchers to determine how participants are finding the study & if the survey URL has been posted elsewhere, &, if needed, request that the link be removed. For example, some websites & apps (e.g., ysense.com, swagbucks.com) post links to research studies that allow users to earn money by completing online surveys. |
| *Data quality checks* | | |
| 45. | Regularly check incoming data. | - Build in data quality checks early & include regular/ongoing checks of the incoming data throughout the lifetime of the survey, incorporating manual review by a member of the research team. This could include reviewing: participant demographics (e.g., age, date of birth), survey responses, survey metadata (e.g., geolocation & IP address, time of survey completion, survey response time). See specific recommendations below. |
|  |  | - For randomised clinical trials, these checks should be completed before randomisation to prevent compromising the randomisation process (Davies et al., 2023). |
|  |  | - Create a clear, pre-defined protocol for handling and communicating with participants flagged as potentially fraudulent, so that potentially genuine participants do not feel their legitimacy is being questioned. |
|  |  | - There is evidence to suggest that fraudulent participants can adjust their tactics or response patterns to evade detection (Storozuk et al., 2020), so manual checks should be completed often. |
| 46. | Manually check for inconsistencies or illogical data, appropriate to the study. | - Review & question any inconsistencies in the information provided (e.g., reporting a current underweight ED diagnosis but providing a weight that is not underweight, or a mismatch between reported age & age based on date of birth). |
|  |  | - Consider prior research with the target population & expected response patterns (e.g., presence of ED symptoms in an ED sample, or binge/compensatory behaviour frequencies with a plausible range). Assess whether participant responses align with these expectations. |
|  |  | - Collect the same information at multiple time points & examine responses any inconsistencies. Also see further information for ‘cross-referenced or duplicate questions’ above. |
| 47. | Manually check for response patterns. | - Do responses follow a pattern? For example, participants may endorse all items positively (e.g., consistently selecting “yes”) or show visual patterns in their responses (e.g., straight-line or diagonal response patterns). |
|  |  | - If a scale includes reverse-scored items (e.g., “I am happy” vs “I am sad”), participants should respond to these items in opposite directions. When they don’t, it may indicate inattention, insufficient effort, or automated responding. |
|  |  | - Check for response patterns both within a single survey & across participants signing up within a short timeframe (e.g., within the same block of time, or on the same day). |
| *Survey metadata* | | |
| 48. | Collect and manually review survey metadata. | - Survey metadata refers to the information that describes a survey, its content, & how it was conducted. This information can include survey completion details (e.g., date/time of submission, response time), participant information (e.g., user language, geolocation, IP address), and other information about the survey’s design, methodology, population, and data quality. Collecting & manually reviewing this data can help to flag potentially fraudulent participants. |
|  |  | - Consider whether to also collect survey paradata (i.e., how participants interact and respond while completing the survey) (Teitcher et al., 2015). This information can include: mouse movements, clicks, or keystrokes; device type or browser used. |
|  |  | - Researchers should evaluate the ethical implications of examining this level of data and decide whether it should be disclosed to participants during informed consent (see Jones et al., 2021). |
| 49. | Manually review time of survey completion. | - Check for suspicious start or completion times relevant to the geographic population & location targeted (e.g., responses completed between the hours of 9pm & 5am). |
|  |  | - Be mindful that genuine participants may complete a survey late at night), so do not rely solely on this strategy; however, this could serve as a red flag. |
|  |  | - There is evidence to suggest that fraudulent participants tend to complete surveys during the middle of the night (Davies et al., 2023; Storozuk et al., 2020). |
| 50. | Manually review survey response time (or speed of survey completion). | - Check if survey response time is significantly faster than expected. For example, surveys completed “too fast” could be a sign of potential fraud. |
|  |  | - A threshold of the mean response time ±2 standard deviations is recommended to indicate potential fraud (Teitcher et al., 2015). |
|  |  | - There is evidence to suggest that fraudulent participants are becoming more effective at completing surveys at realistic speeds to avoid detection (Storozuk et al., 2020), so do not rely solely on this strategy. |
|  |  | - Consider not just the time participants take to complete a survey, but more specifically, certain items or question blocks. For example, long durations to complete a reCAPTCHA block may be an indicator of agentic AI use (see Walker et al., 2026). |
| 51. | Manually review geolocation & IP address. | - Check IP address using a publicly available website such as whatismyipaddress.com to check whether they match recruitment criteria (e.g., is IP address within the expected enrolment geographic location). |
|  |  | - Check for duplicate IP addresses. |
|  |  | - Check whether a VPN has been used (e.g., use of a VPN in addition to other red flags may indicate fraud). |
|  |  | - Researchers should be cautious when drawing conclusions using IP address & should only use this strategy in tandem with multiple other strategies. |
|  |  | - Be mindful that some genuine participants may use the same network or device (e.g., staff using the same WiFi network such as a VPN, participants living in the same household). |
| 52. | Check user language (survey embedded data). | - Check whether recorded user language matches recruitment criteria (e.g., English-language expected). |

*Note*. AI = artificial intelligence; CAPTCHA = revised Completely Automated Public Turing test to tell Computers and Humans Apart; reCAPTCHA = revised CAPTCHA; ED = eating disorder; IP = internet protocol; PPI = patient & public involvement; VPN = virtual private network.

^a^ Several categories may apply to a single strategy based on how it is applied.

**S1.2. References**

**References**

Bowen, A. M., Daniel, C. M., Williams, M. L. & Baird, G. L*.* (2008). Identifying multiple submissions in internet research: Preserving data integrity. *AIDS & Behavior,* *12*, 964–73. <https://doi.org/10.1007/s10461-007-9352-2>

Chandler, J. J., & Paolacci, G. (2017). Lie for a dime: When most prescreening responses are honest but most study participants are impostors. *Social Psychological & Personality Science*, *8*(5), 500–8. <https://doi.org/10.1177/1948550617698203>

Davies, M. R., Monssen, D., Sharpe, H., Allen, K. L., Simms, B., Goldsmith, K. A., Byford, S., Lawrence, V., & Schmidt, U. (2023). Management of fraudulent participants in online research: Practical recommendations from a randomized controlled feasibility trial. *International Journal of Eating Disorders*, 1–11. <http://doi.org/10.1002/eat.24085>

Donegan, K. R., & Gillan, C. M. (2022). New principles and new paths needed for online research in mental health: Commentary on Burnette et al. (2021). *The International Journal of Eating Disorders*, *55*(2), 278–81. <https://doi.org/10.1002/eat.23670>

Dupuis, M., Meier, E. & Cuneo, F. Detecting computer-generated random responding in questionnaire-based data: A comparison of seven indices. *Behavior Research Methods, 51*, 2228–37 (2019). <https://doi.org/10.3758/s13428-018-1103-y>

Hamby, T., & Taylor, W. (2016). Survey satisficing inflates reliability and validity measures: an experimental comparison of college and Amazon Mechanical Turk samples. *Educational & Psychological Measurement*, *76*(6), 912–32. <https://doi.org/10.1177/0013164415627349>

Jones, A., Caes, L., Rugg, T., Noel, M., Bateman, S., & Jordan, A. (2021). Challenging issues of integrity and identity of participants in non-synchronous online qualitative methods*.* ***Methods in Psychology*, *5***, 100072. <https://doi.org/10.1016/j.metip.2021.100072>

Konstan, J. A., Rosser, B. R. S., Ross, M. W., Stanton, J., & Edwards, W. M. (2005). The story of subject naught: A cautionary but optimistic tale of internet survey research*.* ***Journal of Computer-Mediated Communication*, *10***(2), JCMC1029. <https://doi.org/10.1111/j.1083-6101.2005.tb00248.x>

Oppenheimer, D. M., Meyvis, T., & Davidenko, N. (2009). Instructional manipulation checks: Detecting satisficing to increase statistical power*.* ***Journal of Experimental Social Psychology*, *45***(4), 867–72. <https://doi.org/10.1016/j.jesp.2009.03.009>

Roehl, J., & Harland, D. (2022). Imposter participants: Overcoming methodological challenges related to balancing participant privacy with data quality when using online recruitment and data collection. *The Qualitative Report*, *27*(11), 2469–85. <https://doi.org/10.46743/2160-3715/2022.5475>

Simone, M., Cascalheira, C. J., & Pierce, B. G. (2024). A quasi-experimental study examining the efficacy of multimodal bot screening tools and recommendations to preserve data integrity in online psychological research. *American Psychologist*, *79*(7), 956–69. <https://doi.org/10.1037/amp0001183>

Storozuk, A., Ashley, M., Delage, V., & Maloney, E. A. (2020). Got bots? Practical recommendations to protect online survey data from bot attacks. *The Quantitative Methods for Psychology*, *16*(5), 472–81. <https://doi.org/10.20982/tqmp.16.5.p472>

Teitcher, J. E. F., Bockting, W. O., Bauermeister, J. A., Hoefer, C. J., Miner, M. H., & Klitzman, R. L. (2015). Detecting, preventing, and responding to “fraudsters” in internet research: Ethics and tradeoffs. *The Journal of Law, Medicine & Ethics: A Journal of the American Society of Law, Medicine & Ethics*, *43*(1), 116–33. <https://doi.org/10.1111/jlme.12200>

Walker, D. C., Tran, M. P. N., Bizer, G. Y., & Flynn, S. T. (2025). Initial attempts to detect or screen out AI responses prove elusive in the age of agentic AI. International Journal of Eating Disorders, 1–8. <https://doi.org/10.1002/eat.70024>

Wright, K. B. (2005). Researching internet-based populations: Advantages and disadvantages of online survey research, online questionnaire authoring software packages, and web survey services. *Journal of Computer-Mediated Communication*, *10*(3), JCMC1034. <https://doi.org/10.1111/j.1083-6101.2005.tb00259.x>

Yarrish, C., Groshon, L., Mitchell, J. D., Appelbaum, A., Klock, S., Winternitz, T., & Friedman-Wheeler, D. G. (2019). Finding the signal in the noise: Minimizing responses from bots and inattentive humans in online research. *The Behavior Therapist*, *42*(7), 235–42.

Ye, G., Tang, Z., Fang, D., Zhu, Z., Feng, Y., Xu, P., Chen, X., & Wang, Z. (2018). Yet another text captcha solver: A generative adversarial network based approach. In Proceedings of the 2018 ACM SIGSAC Conference on Computer and Communications Security (CCS '18), Toronto, Canada, 332–48. Association for Computing Machinery. <https://doi.org/10.1145/3243734.3243754>
